# Supplementary material for: Risk of Early Childhood Obesity in Offspring of Women with Preeclampsia: A Population-Based Study
Source: J Clin Med. 2021 Aug 23;10(16):3758. doi: 10.3390/jcm10163758 (PMC8397009; doi:10.3390/jcm10163758)
Supplement: Supplementary file 1 [file jcm-10-03758-s001.zip › jcm-1322258-supplementary.pdf]

Journal of
Clinical Medicine

MDPI

Supplementary Material

Supplementary Table S1. ICD-10 Code for pre-pregnancy hypertension and pre-pregnancy diabetes mellitus.

	Code	Description
Pre pregnancy hypertension	I10	Essential (primary) hypertension
	I11	Hypertensive heart disease
	I12	Hypertensive chronic kidney disease
	I13	Hypertensive heart and chronic kidney disease
	I15	Secondary hypertension
Pre pregnancy diabetes	E10	Type 1 diabetes mellitus
	E11	Type 2 diabetes mellitus
	E12	Malnutrition-related diabetes mellitus
	E13	Other specified diabetes mellitus
	E14	Unspecified diabetes mellitus

Supplementary Table S2. Body mass index of male offspring up to 80 months of age stratified according to maternal preeclampsia (PE).

Age	Offspring born to women without PE	Offspring born to women with PE	*p*
30–42 months	16.12 (15.33-16.99)	16.11 (15.29-17.00)	0.1946
42–54 months	15.99 (15.21-16.84)	16.01 (15.19-16.96)	0.0367
54–66 months	15.87 (15.06-16.81)	15.94 (15.07-16.98)	<0.0001
66–80 months	15.89 (15.01-17.03)	16.07 (15.06-17.47)	<0.0001

Supplementary Table S3. Body mass index of female offspring up to 80 months of age stratified according to maternal preeclampsia (PE).

Age	Offspring born to women without PE	Offspring born to women with PE	*p*
30–42 months	15.87 (15.06-16.75)	15.83 (14.99-16.76)	0.0063
42–54 months	15.81 (15.02-16.69)	15.82 (14.96-16.73)	0.5986
54–66 months	15.67 (14.88-16.66)	15.78 (14.87-16.81)	<0.0001
66–80 months	15.72 (14.84-16.82)	15.83 (14.84-17.10)	<0.0001

Supplementary Table S4. Prevalence and associations between maternal history of preeclampsia (PE) and underweight and obese male offspring until 80 months of age.

Age	Offspring born to women without PE	Offspring born to women with PE	*p*	Unadjusted OR (95% CI)	Adjusted* OR (95% CI)
Obesity					
30–40 months	39752/507959 (7.8%)	886/9593 (9.2%)	<0.0001	1.19(1.11-1.28)	1.31 (1.22-1.41)
42–54 months	57064/494254 (11.5%)	1352/9477 (14.3%)	<0.0001	1.27(1.20-1.35)	1.36 (1.28-1.45)
54–66 months	54775/458204 (12.0%)	1382/8901 (15.5%)	<0.0001	1.35(1.27-1.43)	1.38 (1.30-1.47)
66–80 months	41310/322135 (12.8%)	1071/6107 (17.5%)	<0.0001	1.44(1.35-1.54)	1.42 (1.33-1.52)
Underweight					
30–40 months	48436/507959 (9.5%)	1081/9593 (11.3%)	<0.0001	1.20(1.13-1.28)	0.98 (0.925-1.05)
42–54 months	35779/494254 (7.2%)	819/9477 (8.6%)	<0.0001	1.21(1.12-1.30)	0.97 (0.907-1.05)
54–66 months	33988/458204 (7.4%)	741/8901 (8.3%)	0.0012	1.13(1.05-1.22)	0.94 (0.872-1.02)
66–80 months	28126/322135 (8.7%)	582/6107 (9.5%)	0.0286	1.10(1.01-1.20)	0.93(0.857-1.02)

Supplementary Table S5. Prevalence and associations between maternal history of preeclampsia (PE) and underweight and obese female offspring until 80 months of age.

Age	Offspring born to women without PE	Offspring born to women with PE	*p*	Unadjusted OR (95% CI)	Adjusted* OR (95% CI)
Obesity					
30–40 months	37950/477033 (8.0%)	855/9663 (8.8%)	0.0013	1.12(1.05-1.20)	1.24(1.15-1.33)
42–54 months	56234/464249 (12.1%)	1351/9566 (14.1%)	<0.0001	1.19(1.13-1.27)	1.30(1.23-1.38)
54–66 months	56640/432240 (13.1%)	1456/8985 (16.2%)	<0.0001	1.28(1.21-1.36)	1.35(1.27-1.43)
66–80 months	40607/304450 (13.3%)	1107/6297 (17.6%)	<0.0001	1.39(1.30-1.48)	1.41(1.32-1.51)
Underweight					
30–40 months	49007/477033 (10.3%)	1139/9663 (11.8%)	<0.0001	1.17(1.10-1.24)	0.93(0.87-0.99)
42–54 months	34141/464249 (7.4%)	899/9566 (9.4%)	<0.0001	1.31(1.22-1.40)	1.03(0.96-1.11)
54–66 months	31364/432240 (7.3%)	750/8985 (8.3%)	<0.0001	1.16(1.08-1.26)	0.94(0.87-1.01)
66–80 months	23758/304450 (7.8%)	589/6297 (9.4%)	<0.0001	1.22(1.12-1.33)	1.01(0.92-1.10)
